# Recent Advances in Modeling, Control and Motion Planning for AI-Driven Soft Robotic Systems

**DOI:** 10.3390/biomimetics11100728

**Published:** 2026-10-11

**Authors:** Lingli Tang, Wenguang Yang, Wenyuan Chen, Jia Yang

**Affiliations:** 1 School of Electrical Engineering and Automation, Tianjin University of Technology, Tianjin 300384, China; tllbestluckyrich@stud.tjut.edu.cn; 2 Tianjin Key Laboratory of New Energy Power Conversion, Transmission and Intelligent Control, Tianjin University of Technology, Tianjin 300384, China; 3 School of Electromechanical and Automotive Engineering, Yantai University, Yantai 264005, China; yangwenguang@ytu.edu.cn; 4 Tianjin Key Laboratory for Advanced Mechatronic System Design and Intelligent Control, School of Mechanical Engineering, Tianjin University of Technology, Tianjin 300384, China; 5 National Demonstration Center for Experimental Mechanical and Electrical Engineering Education, School of Mechanical Engineering, Tianjin University of Technology, Tianjin 300384, China; 6 State Key Laboratory of Robotics and Intelligent Systems, Shenyang Institute of Automation, Chinese Academy of Sciences (CAS), Shenyang 110016, China

**Keywords:** soft robots, artificial intelligence, system modeling, intelligent control, motion planning

## Abstract

Due to their high compliance, environmental adaptability, and inherent safety in human–robot interaction, soft robots have demonstrated considerable potential, including medical rehabilitation, compliant grasping, and operations in complex environments. Unlike conventional robots relying on rigid structures and discrete joint motions, soft robots use flexible materials such as elastomers and hydrogels to achieve motion and functionality through continuous deformation. Despite providing excellent adaptability, these compliant materials also introduce complex behaviors, including continuous morphological changes, material nonlinearities, time-varying properties, and environmental coupling, posing substantial challenges to modeling, control, and motion planning. In recent years, artificial intelligence (AI), through nonlinear representation, data-driven learning, adaptive optimization, and autonomous decision-making, has provided new technical pathways for addressing these challenges. Unlike previous reviews organized primarily by AI algorithm type, this work narratively reviews 39 studies and organizes AI-driven approaches according to the soft robotic system implementation process, encompassing modeling, control, and motion planning. Specifically, in modeling, neural networks, Koopman representations, and physics–data fusion methods have enhanced the representation of complex dynamical relationships. In control, learning-augmented control and reinforcement learning have reduced reliance on accurate system models and improved adaptability under uncertainty. In motion planning, learning from demonstration, reinforcement learning, and intelligent optimization methods have strengthened the capability for autonomous motion generation under complex constraints. Finally, this paper summarizes the current state of the field and discusses future trends in AI-driven soft robotics, emphasizing stronger coordination across functional modules. Closed-loop integration of prediction, decision-making, control, and feedback updates may further advance soft robots from stage-specific intelligence toward system-level autonomy.

## 1. Introduction

**Figure 1:**
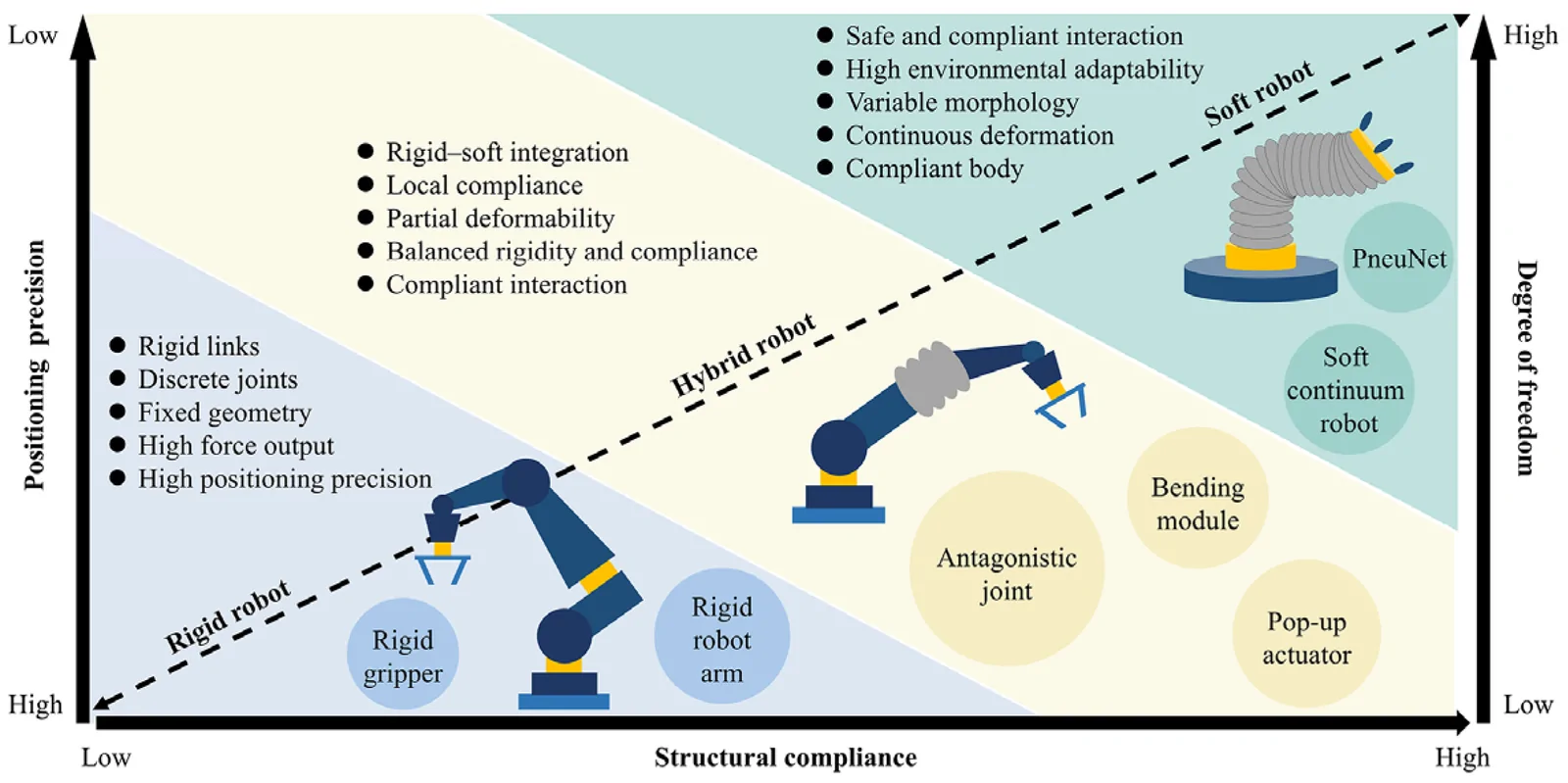
Comparison of material stiffness and performance characteristics of soft, hybrid, and rigid robots.

**Figure 2:**
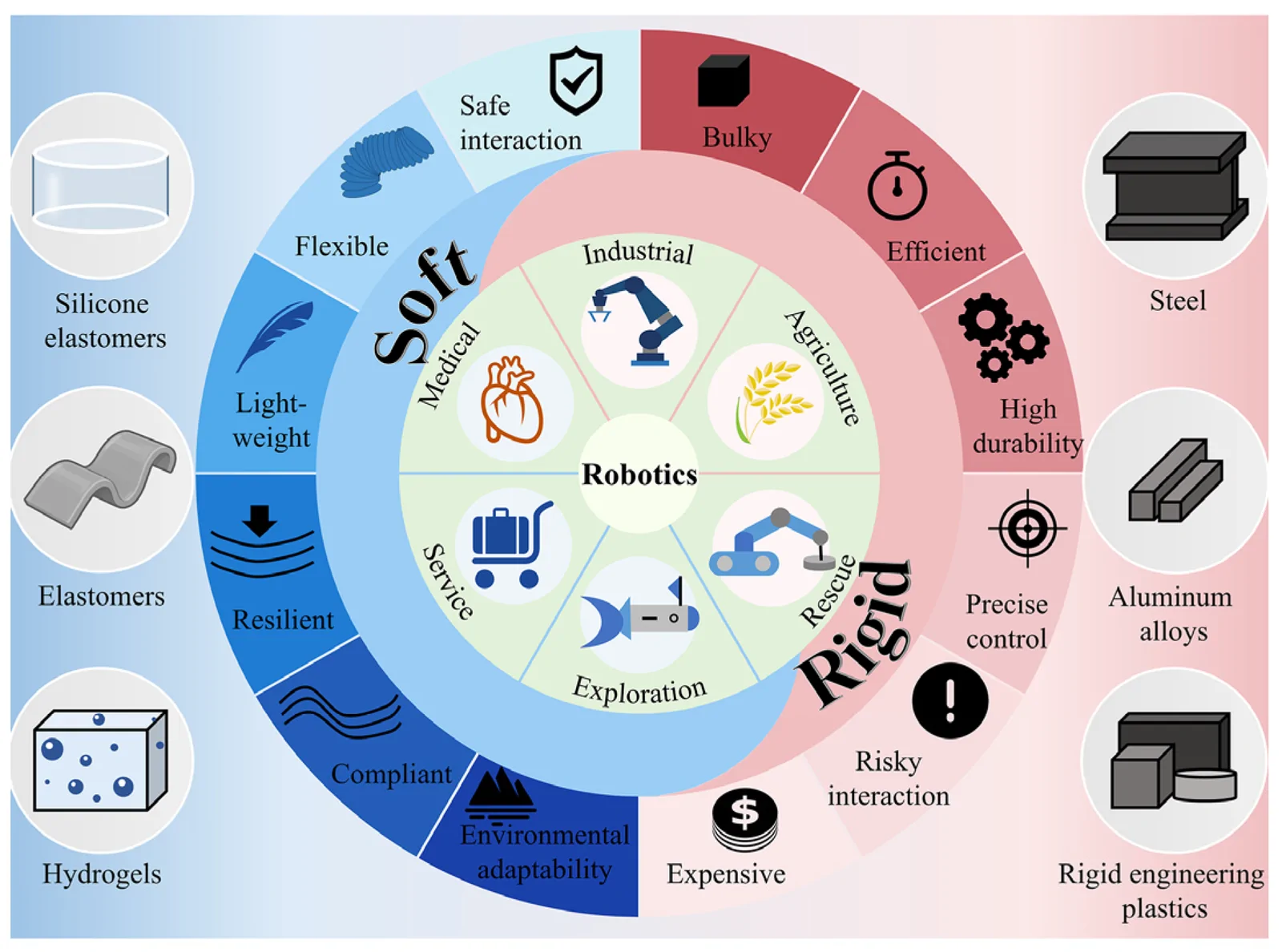
A schematic comparison of material features, merits and demerits, and application scenarios for soft and rigid robotic systems.

With the rapid development of robotics, advanced manufacturing, and artificial intelligence, robotic systems have been widely applied in industrial manufacturing, medical rehabilitation, service robotics, and human–robot collaboration [1]. As illustrated in Figure 1 and Figure 2, soft and rigid robots differ substantially in material stiffness, structural compliance, degrees of freedom, and interaction characteristics, resulting in distinct advantages and limitations in robotic applications.

Rigid robots typically consist of rigid links and joints and rely on accurate kinematic and dynamic models to achieve high-precision manipulation, exhibiting excellent stability and repeatability in structured environments [2,3]. However, as robotic applications increasingly extend into complex environments and scenarios involving direct physical interaction with humans, the limitations of rigid robots in environmental adaptability, safety, and compliant interaction have become increasingly evident [4].

To address these limitations, soft robotic systems have emerged as a new robotic paradigm and attracted considerable attention [4,5]. Characterized by high compliance and intrinsic safety, soft robots demonstrate excellent capabilities in human–robot interaction, demonstrating substantial application potential in soft grasping, wearable devices, biomedical applications, and rehabilitation assistance [2,6,7].

It should be noted that the distinctive compliance of soft robots, while enhancing their adaptability to the environment, also poses considerable engineering challenges. Soft robots are typically characterized by continuum structures, complex actuation mechanisms, and strongly nonlinear material properties, with their motion often involving large deformations, material hysteresis, and strong coupling with the environment [8,9]. Unlike rigid robots, whose motion states can generally be represented by a finite set of joint variables, soft robots behave as continuously deformable compliant bodies, making their shape variations difficult to characterize accurately using a finite set of parameters [10,11,12]. Consequently, conventional rigid-body dynamic models cannot be directly applied to soft robotic systems. The resulting modeling uncertainties not only complicate state estimation and controller design but also undermine the reliability of motion planning in complex environments [13]. Therefore, accurate modeling, stable control, and autonomous motion planning remain key challenges to the practical deployment of soft robotic systems.

In recent years, advances in AI have provided new avenues for addressing these challenges. For example, machine learning and deep learning methods can learn complex nonlinear dynamic relationships in soft robotic systems from experimental or simulation data, thereby supporting data-driven modeling and state estimation, whereas reinforcement learning can autonomously optimize control policies through interactions with the environment to accomplish complex tasks [11,14,15]. AI-driven approaches are therefore increasingly regarded as promising means of overcoming key challenges in soft robot modeling, control, and motion planning. Accordingly, this review focuses on recent advances in AI-driven approaches for soft robotic systems and discusses their future research directions.

Compared with previous reviews, this work makes three main contributions. First, AI-driven approaches are organized according to the soft robotic system implementation process, which includes modeling, control, and motion planning, rather than by AI algorithm type. Second, recent advances are evaluated using quantitative performance metrics in addition to summarizing methods and applications. Third, future prospects for collaborative optimization across modeling, control, and motion planning are discussed. Accordingly, Section 2 presents the review methodology, followed by reviews of AI-driven modeling, control, and motion planning, and finally a discussion of future prospects and conclusions.

## 2. Review Methodology

### 2.1. Search Strategy

A comprehensive literature search was performed using major academic databases, including Google Scholar and Web of Science, to retrieve relevant publications from 2015 to 2026. The search strategy was designed by combining terms related to soft robotics, artificial intelligence techniques, and robotic system-level tasks. The Boolean search string was formulated as follows: (“soft robot” OR “continuum robot” OR “soft robotic system”) AND (“artificial intelligence” OR “deep learning” OR “neural network”) AND (“modeling” OR “control” OR “path planning”).

### 2.2. Screening and Filtration Process

The retrieved studies were further screened and filtered according to predefined inclusion criteria, including publication year, research subject, research domain, and research methodology. These criteria were established to ensure that only studies directly related to AI-driven soft robotic systems were included in the final review. The inclusion and exclusion criteria used for literature selection in this review are summarized in Table 1.

### 2.3. Review Scope and Core Contributions

Material properties, continuum structures, actuation–structure coupling, and environmental interactions jointly affect the modeling, control, and motion planning of soft robots. Material nonlinearities and time-varying behaviors increase model uncertainty; continuous deformation and high degrees of freedom complicate shape representation; actuation–structure coupling leads to complex input–deformation relationships; and environmental interactions further increase dynamic uncertainty. Together, these factors constitute key challenges for applying AI to soft robotic systems.

In recent years, AI technologies have advanced rapidly in soft robotics. However, existing reviews have primarily focused on specific AI algorithms or individual robotic tasks, with limited integrated analysis from the perspective of the overall soft robotic system development process. Existing reviews have addressed the modeling and control of soft robots from different perspectives. For example, Thuruthel et al. reviewed learning-based control approaches for soft robots [11], Wang et al. focused on machine learning-based control of continuum robots [15], and Della Santina et al. reviewed model-based control approaches [8]. The Advances in Modelling and Control of Soft Robots Research Topic covers state-variable modeling and reconstruction [16], dynamic modeling and adaptive predictive control [17], and model predictive control [18] for soft robotic systems. While these studies have contributed substantially to advances in soft robot modeling and control, motion planning has received comparatively limited attention as an independent stage alongside modeling and control. Therefore, this review adopts an application-oriented framework organized according to the development process of soft robotic systems, spanning modeling, control, and motion planning. It narratively summarizes recent applications of AI-driven approaches across these key stages and analyzes their roles in addressing the challenges associated with nonlinear modeling, complex control, and environmental adaptation.

This review is guided by the following research questions:

1. How are different AI approaches applied to modeling, control, and motion planning of soft robots, and what key technical challenges do they address?
2. Compared with conventional methods, what improvements do AI-driven approaches provide in terms of prediction accuracy, computational efficiency, control stability, and adaptability?
3. How can modeling, control, and motion planning modules be jointly optimized to advance intelligent and autonomous soft robotic systems?

Compared with previous reviews, the contributions of this work are threefold. First, rather than classifying methods by AI algorithms or control strategies, this review organizes AI applications around the soft robotic system workflow, covering modeling, control, and motion planning. Second, beyond summarizing AI methods and applications, this review identifies recent advances using quantitative metrics, including prediction error, positioning accuracy, and computational time, and presents them in tabular form. Finally, this review discusses future prospects for multi-module collaborative optimization among modeling, control, and motion planning, providing insights into the development of intelligent and autonomous soft robotic systems.

## 3. Challenges in the Research of Soft Robot Systems

Soft robotic systems commonly employ elastomers such as silicone, rubber, and polyurethane as primary structural materials, which can be fabricated into complex compliant structures and internal fluidic channels through techniques such as molding, soft lithography, and additive manufacturing [5,19]. Hydrogels, electroactive polymers, shape-memory materials, liquid crystal elastomers, and magnetically responsive composites have also been used to construct soft actuators that respond to electrical, thermal, optical, magnetic, or chemical stimuli [20]. In addition, fabrics, fiber-reinforced composites, granular-jamming structures, and low-melting-point-alloy-based mechanisms can be employed to regulate deformation and modulate system stiffness [4,21,22]. Collectively, these diverse material systems and stiffness-modulation strategies provide the material and technological basis for the compliant deformation of soft robotic systems.

In contrast to rigid robots composed primarily of metals and rigid engineering plastics, the materials used in soft robotic systems are generally characterized by low stiffness, large deformation capability, and continuous deformability. Motion generation in soft robots relies primarily on the deformation of their constituent materials and structures, rather than on discrete joint motion between rigid links [10,23]. These characteristics enable soft robots to passively conform to the shapes of objects and environments, reduce collision impacts, and achieve enveloping grasping, navigation through confined spaces, and safe human–robot interaction [13,24]. However, these same material characteristics may also lead to limited load-bearing capacity, high sensitivity of robot configurations to external loads, as well as pronounced nonlinearities, hysteresis, and parameter uncertainty [4,5]. Thus, soft materials not only underpin the compliance and environmental adaptability of soft robotic systems but also introduce substantial challenges for their modeling, control, and motion planning. The following sections discuss these challenges from the perspectives of modeling, control, and motion planning, respectively.

It should be noted that learning-related challenges are not set as a separate category in this review, because learning is not an independent stage of soft robotic system implementation but is embedded throughout modeling, control, and motion planning. Accordingly, learning-specific challenges, including data requirements, training efficiency, generalization, interpretability, and stability and safety guarantees, are incorporated into the corresponding discussions of these three technical dimensions.

### 3.1. Modeling Challenges

As discussed above, soft robots are typically composed of low-modulus materials such as silicone rubber and elastomers [25], and their motion primarily relies on the continuous deformation of materials and structures rather than the discrete rotation and translation of rigid links [26]. Unlike rigid robots, whose configurations can be described by a finite number of joint variables, soft robots theoretically possess infinite degrees of freedom, with bending, twisting, stretching, and shearing potentially occurring at any point along their bodies [27,28]. Consequently, a fundamental challenge in the modeling of soft robotic systems lies in representing their continuous configurations with a finite set of state variables. Webster and Jones pointed out that continuum robots are commonly modeled under the Piecewise Constant Curvature (PCC) assumption, in which the continuous structure is approximated by a sequence of constant-curvature segments, thereby establishing mappings among actuator, configuration, and task spaces [29]. This approach can substantially reduce the dimensionality of the model; however, its accuracy relies on assumptions such as constant curvature, homogeneous material properties, and ideal actuation. Under the influence of gravity, external loading, or spatially nonuniform actuation, these assumptions may no longer hold, and the robot may exhibit variable-curvature deformation. Gazzola et al. employed Cosserat rod theory to describe coupled bending, twisting, stretching, and shearing deformations in slender soft structures, while also accounting for contact, friction, and fluid interactions, highlighting that the global configuration of soft structures is generally governed by multiple, strongly coupled deformation modes [30]. Therefore, a primary modeling challenge for soft robots is to reduce high-dimensional continuous deformation to an identifiable and computationally tractable low-dimensional state representation while preserving the dominant geometric and mechanical characteristics.

After establishing an appropriate configuration representation, soft robots still require models that capture the relationships among actuation inputs, material stresses, and structural deformations. Under large strains, the coupling between material and geometric nonlinearities makes conventional small-deformation linear models inadequate. To characterize the large-deformation behavior of soft materials, various hyperelastic constitutive models have been developed, including models based on strain-energy functions for incompressible materials [31], the eight-chain model derived from polymer molecular-chain networks [32], and strain-energy formulations based on the first strain invariant [33]. These models provide important foundations for describing nonlinear material responses under different loading conditions.

However, existing constitutive models differ in their theoretical assumptions, parameter requirements, and applicable deformation ranges. Their parameters typically require experimental identification and are sensitive to loading paths, specimen characteristics, and material variations. Consequently, uncertainties in model selection and parameter identification remain important sources of error in predicting the pose and output force of soft robots. Moreover, Moseley et al. demonstrated that the deformation response of soft pneumatic actuators is jointly affected by internal pressure, material properties, structural geometry, and boundary conditions [34].

Beyond the instantaneous nonlinearities caused by large deformation, compliant materials also exhibit pronounced time- and history-dependent behaviors, including rate effects, creep, stress relaxation, hysteresis, and the Mullins effect. Previous studies have characterized these phenomena using constitutive models that account for viscoelasticity and cyclic-loading effects. For example, elastomers exhibit distinct transient responses and hysteresis under different loading rates and cyclic loading conditions [35]; internal-variable-based approaches have been developed to describe viscous evolution under finite deformation [36]. Cyclic loading can induce stress softening phenomena [37]. However, such models generally require internal state variables that are difficult to measure directly and additional parameters that must be identified experimentally. Furthermore, fatigue, aging, and batch-to-batch variations in material properties can progressively alter the elastic modulus, damping, and hysteretic characteristics, making fixed-parameter models calibrated under a limited set of experimental conditions difficult to maintain predictive accuracy across varying loading histories and prolonged operation.

The overall response of soft robots is further complicated by strong coupling among actuation systems, compliant structures, and external environments. During operation, soft structures may simultaneously experience external forces such as gravity, contact, friction, and fluid forces, while undergoing coupled bending, twisting, stretching, and shearing deformations [30]. In practical tasks, continuous interaction with the environment can dynamically alter contact conditions and boundary constraints, giving rise to time-varying and nonsmooth system dynamics. Consequently, modeling actuation inputs or structural deformation in isolation is generally insufficient to accurately predict the behavior of soft robots. Accurate modeling therefore requires an integrated representation of the dynamic coupling among actuation, material properties, structural geometry, boundary conditions, and environmental interactions.

In summary, soft robot modeling is challenged by the combined effects of continuous deformation, nonlinear and time-dependent material behavior, and coupling among actuation, structure, and environmental interactions. Physics-based models can capture these effects at different levels of fidelity, but more detailed models may require additional parameter identification and computational resources, whereas simplified models may neglect important physical effects. Therefore, a key challenge is to balance physical consistency, prediction accuracy, computational efficiency, and adaptability while accounting for variations in material properties, parameter drift, and changing environmental interactions.

### 3.2. Controlling Challenges

While compliant materials endow robots with desirable compliance, intrinsic safety, and environmental adaptability, they also increase control challenges at multiple stages, including control input generation, system response, state feedback, and environmental interaction [38,39]. Unlike rigid robots with well-defined joints and relatively well-characterized dynamic parameters, the motion of soft robots relies on the continuous deformation of compliant structures [40,41]. Their control performance is therefore determined not only by actuation inputs, but also by material deformation history, the deformation state, and external contact conditions. Accordingly, the control challenges induced by compliant materials can be summarized within the closed-loop control framework as follows [42].

The mismatch between distributed deformation and limited actuation makes it difficult to accurately map desired motions to control inputs. Thuruthel et al. noted that the high-dimensional continuous state space and nonlinear dynamics of soft manipulators complicate the development of general control frameworks [11]. Giorelli et al. found that tendon-driven soft manipulators with non-constant curvature exhibit complex nonlinear multi-input mappings between actuation inputs and end-effector poses, making conventional analytical inverse kinematics difficult to apply directly [43]. Similarly, Rolf and Steil showed that conventional analytical methods struggle to accurately establish the inverse mapping from desired end-effector positions to actuation inputs for a bio-inspired elephant-trunk continuum robot [44]. Therefore, establishing a stable, accurate, and transferable motion-to-actuation mapping remains a major challenge for precise control of soft robots.

The nonlinear, viscoelastic, hysteretic, and time-varying properties of compliant materials reduce the consistency of soft actuator responses to control commands. For example, Pneumatic Artificial Muscles (PAMs) exhibit significant static and dynamic nonlinearities among internal pressure, contraction length, and output force [45]. Tondu and Lopez further showed that fiber friction, structural deformation, and material nonlinearities in McKibben pneumatic artificial muscles can induce significant hysteresis [46]. Rolf and Steil also found that actuation range, sensor response, and robot morphology may vary with operating conditions, making fixed control gains and compensation parameters difficult to maintain over extended operation [44]. These material and system characteristics make stable and repeatable motion control more difficult to achieve.

The distributed states of compliant structures are difficult to observe accurately, limiting the error compensation capability of closed-loop feedback. The curvature, strain, contact forces, and overall configuration of soft robots are distributed throughout their continuously deforming bodies and are difficult to capture directly using a limited number of fixed sensors. Zhao et al. embedded stretchable optical waveguides into a soft prosthetic hand to sense bending, elongation, and contact, highlighting the importance of compliant sensing approaches that deform with soft structures for state sensing [47]. Truby et al. integrated sensing elements into soft actuators through embedded 3D printing, enabling proprioceptive and haptic feedback [48]. Furthermore, Thuruthel et al. combined redundant soft sensors with inference methods to estimate robot configuration, external forces, and contact states, indicating that raw sensory signals generally require further state estimation before being used for closed-loop control [49]. Therefore, obtaining accurate and stable state feedback remains essential for improving the accuracy and robustness of closed-loop control.

Environmental interactions continuously alter the boundary conditions and loading states of soft robots, increasing the difficulty of maintaining closed-loop stability. Della Santina et al. incorporated trajectory tracking and robot–environment interaction into a unified dynamic feedback control framework, showing that stable contact control requires joint consideration of structural compliance, robot configuration, and external forces [50]. Homberg et al. found that complex structural deformations induced by contact are difficult to directly measure or estimate, complicating contact-state sensing and precise control [51]. Li et al. further showed that transient large deformations induced by collisions impose greater demands on state recovery and control stability [52]. Therefore, environmental interaction introduces dynamic coupling among contact forces, structural deformation, and actuation responses, substantially increasing the difficulty of trajectory maintenance, contact-force regulation, and closed-loop stability control.

In summary, the challenges of soft robot control span the closed-loop process, involving actuation, state feedback, and environmental interaction. Continuous deformation and high-dimensional configurations, often combined with underactuation, complicate the coordination of control inputs. Material nonlinearities, hysteresis, and time-varying properties make actuator responses difficult to predict, while limited state sensing constrains feedback-based compensation. Environmental contact further introduces varying boundary conditions and external disturbances. These interrelated challenges complicate the achievement of accurate, stable, and adaptive closed-loop control under model uncertainty, limited state information, and changing environmental conditions. Conventional control approaches that rely on accurate models, fixed parameters, or predefined control laws may face limitations under such conditions. Data-driven control, intelligent optimization, and reinforcement learning offer potential approaches to addressing these challenges and improving the control performance and adaptability of soft robotic systems.

### 3.3. Planning Challenges

Compliant materials endow soft robots with intrinsic compliance and environmental adaptability [53], but they also make motion planning a more complex problem beyond conventional end-effector trajectory planning for rigid robots, as whole-body shape variations must be considered [54]. Because soft robots undergo continuous deformation, both the end-effector pose and overall morphology evolve dynamically in response to actuation, loading, and environmental interaction [55]. Consequently, conventional planning methods based solely on end-effector trajectories and fixed geometric models cannot reliably guarantee the safety of whole-body motion [56,57]. Marchese et al. demonstrated that the high compliance of soft robots makes it difficult for conventional planning methods to account for whole-body shape variations. In confined environments, planning only the motion of the end effector can lead to collisions involving other portions of the robot, thereby requiring planning strategies that explicitly account for the overall robot shape [58]. Godage et al. further showed that the configuration of multi-section continuum robots is difficult to predict accurately, while limited sensory information restricts the estimation of internal states and environmental relationships, complicating obstacle-avoidance planning [59].

In addition to whole-body shape variation, the high dimensionality and kinematic redundancy associated with compliant structures further increase the complexity of motion-planning search. Continuum robots theoretically possess an infinite number of degrees of freedom, resulting in a configuration space that is substantially higher-dimensional than that of rigid robots and requiring planners to simultaneously satisfy motion continuity, shape, and environmental constraints. Wu et al. showed that hyper-redundancy substantially increases the complexity of path search, making it difficult for conventional methods to maintain computational efficiency while satisfying global collision-avoidance constraints [60]. Niu et al. demonstrated that discrete paths generated by conventional sampling-based methods cannot be directly applied to continuum robots and require further path fitting before execution, potentially introducing additional collision risks [61]. Chen et al. further emphasized that motion planning for compliant continuum robots must simultaneously address high-dimensional configuration spaces, multiple motion constraints, and shape continuity constraints, making searching for feasible paths substantially more challenging [62].

Moreover, compliant materials introduce substantial configuration uncertainty, posing significant challenges for obstacle-avoidance planning in complex environments. Under the combined effects of gravity, actuation, and environmental interactions, robot configurations can vary continuously, making robot–obstacle separation difficult to predict accurately. Fairchild et al. highlighted that path planning for soft manipulators in obstacle-filled environments must account for dynamic variations in the robot’s global shape, making conventional obstacle-avoidance methods based on fixed geometric boundaries difficult to apply directly [63]. Lai et al. further showed that axial compression, variable stiffness, and coupled deformation among multiple sections induced by compliant structures can alter the robot’s actual configuration, requiring path planning to simultaneously consider shape variation and spatial constraints [64]. Niu et al. also noted that deviations between the actual configuration of a compliant robot and its nominal trajectory can hinder accurate execution in complex and constrained environments [61]. Owing to continuous deformation and complex shape variations caused by compliant materials, multi-section continuum robots must simultaneously satisfy configuration continuity constraints and motion constraints during path generation, making it difficult for conventional planning methods to directly obtain feasible and executable trajectories [65,66].

Overall, the continuous deformation arising from structural compliance gives rise to three key challenges in soft robot motion planning. First, deformation uncertainty resulting from interactions between compliant structures and the environment makes collision constraints and obstacle-avoidance conditions difficult to characterize accurately, thereby reducing planning reliability in complex environments. Second, the high dimensionality and kinematic redundancy inherent in continuum structures substantially enlarge the configuration space that must be explored, making the efficient search and optimization of feasible paths more challenging. Third, the robot’s whole-body shape varies dynamically in response to actuation and environmental interactions, making its global configuration difficult to predict accurately and further increasing the complexity of whole-body motion planning. Consequently, motion planning for soft robots extends beyond conventional geometric path generation and becomes a high-dimensional adaptive planning problem that must simultaneously account for shape variation, spatial constraints, and environmental interactions. These challenges motivate the development of AI-driven approaches based on learning, optimization-based search, and autonomous decision-making to enhance motion-planning capabilities in complex and uncertain environments.

**Figure 3:**
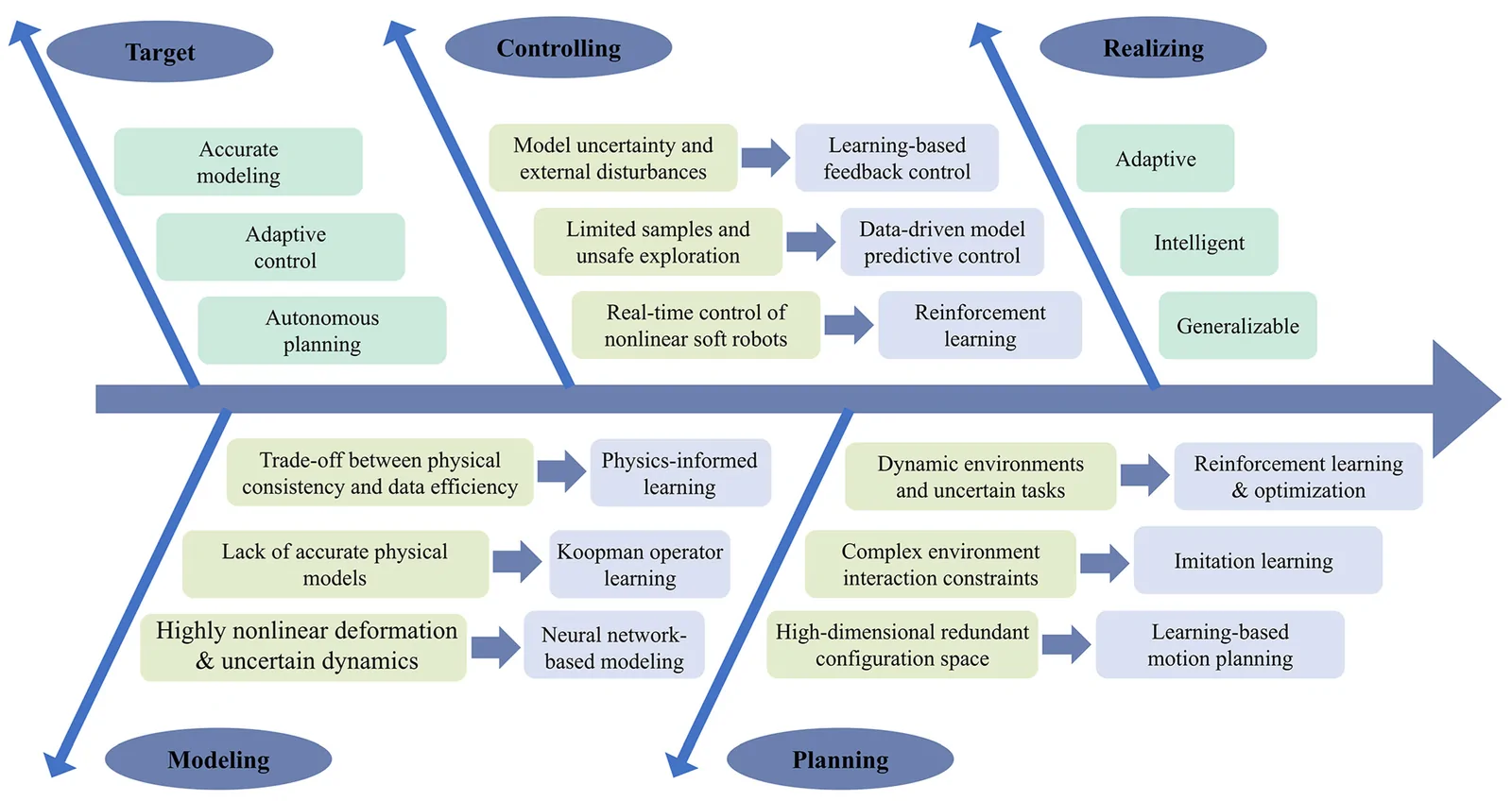
Overview of the key challenges, AI-driven solutions, and ultimate goals in the modeling, control, and planning of soft robots.

The preceding sections have laid out the core challenges encountered in the modeling, control, and motion planning of soft robots, which are summarized in Table 2. As illustrated in Figure 3, each of these three technical dimensions is characterized by unique key challenges, potential AI-driven solutions, and ultimate research goals. Building on this overview of bottlenecks and target objectives, the following sections review state-of-the-art advances for modeling, control, and motion-planning in sequence.

## 4. AI-Driven Methods for Addressing Modeling Challenges in Soft Robotic Systems

Reliable system models provide an essential foundation for state prediction, motion control, and task planning in soft robotic systems. Previous studies have demonstrated how different modeling strategies can support soft robot control. Thuruthel et al. employed a reduced first-order dynamic model to facilitate closed-loop task-space control [67], Chen et al. developed a state-change model for feedforward control and external load estimation [68], and Gillespie et al. learned nonlinear dynamics using deep neural networks for model predictive control [69]. However, continuous deformation, material nonlinearities, and complex coupling effects pose substantial challenges to conventional analytical modeling and parameter identification [10]. Consequently, current research has gradually shifted from purely physics-based models toward data-driven and hybrid physics-data-driven modeling approaches [70]. AI methods provide powerful nonlinear representation and data-fitting capabilities, enabling these approaches to learn complex relationships between actuation inputs and robot configurations [71,72]. Moreover, their capabilities for temporal feature extraction and dynamic representation learning allow hysteresis, dynamic coupling, and temporal dependencies to be effectively captured. In addition, incorporating physical priors into data-driven models enables more effective representation of high-dimensional, coupled, and uncertain system dynamics [73]. Therefore, AI-driven approaches are increasingly enabling improvements in predictive accuracy, computational efficiency, and adaptability of soft robotic systems under complex operating conditions.

### 4.1. Inverse Model Learning Based on Neural Networks

Early data-driven studies focused on avoiding the computational complexity of solving analytical inverse kinematics or inverse statics models and directly learning the mapping between desired robot configurations and actuation inputs [74,75]. For soft robots, factors such as nonconstant curvature, structural redundancy, friction, and fabrication errors make the development and solution of conventional inverse models particularly challenging [76]. As system nonlinearities and mapping complexity increase, shallow data-fitting methods also become increasingly limited in representing complex relationships. Neural networks have therefore been adopted to learn more complex inverse mappings.

Giorelli et al. employed a feedforward neural network to learn the inverse static mapping of a cable-driven soft manipulator, addressing the difficulty of obtaining accurate solutions using conventional analytical methods in the presence of nonconstant curvature and model uncertainties. Experimental results showed that, in terms of accuracy, the neural network achieved a mean end-effector positioning error of 7.35 mm, substantially lower than the 15.12 mm obtained with the Jacobian method. Moreover, 91% of the test samples produced errors below 15 mm with the neural network, compared with only 43.18% for the Jacobian method. In terms of computational efficiency, the Jacobian method required approximately 12 iterations on average and about 246 ms for each solution, whereas a single prediction using the neural network required only 0.114 ms. These results highlight the capability of neural networks to approximate complex nonlinear inverse mappings while improving positioning accuracy and substantially reducing computational cost [43]. Beyond static inverse mappings, subsequent studies extended neural-network-based modeling to time-dependent soft-robot dynamics associated with material hysteresis, parameter variations, and dynamic coupling. For example, Melingui et al. developed a two-level neural network framework for a continuum robot characterized by strong nonlinearities, time-varying behavior, and hysteresis, combining kinematic learning with adaptive dynamic identification. Within this framework, the kinematic level employed a multilayer neural network to learn the mapping between end-effector positions and actuation variables, whereas the dynamic level used a modified Elman neural network to identify the system dynamics and adaptively update the model online. Experimental results demonstrated accurate end-effector trajectory tracking and good adaptability to variations in system dynamics, indicating that neural-network-based models can be further extended to the representation of dynamic behaviors involving temporal dependencies and memory effects [77].

As application environments become increasingly complex, data-driven models have gradually evolved from representing the intrinsic characteristics of robots toward adapting to robot-environment interactions. Lee et al. employed a nonparametric online learning framework based on locally weighted projection regression to establish an inverse kinematic mapping and initialized the model using finite element analysis data to compensate for model mismatch. These results demonstrate that online learning can effectively improve the adaptability of data-driven inverse models under unknown environmental interactions [78]. Building on this development, the state representation used in data-driven modeling was further extended from low-dimensional end-effector variables to the whole-body shape of the robot. Almanzor et al. employed deep convolutional neural networks to learn an inverse kinematic mapping based on a visual representation of the robot’s whole-body shape, mapping the desired shape, current shape, and current actuation state to the next actuation command. This approach addresses the limitation of low-dimensional state representations in accurately representing soft robot configurations. In experiments on a physical robot, the average image mask error between the desired and achieved shapes decreased from 0.0354 to 0.0133, corresponding to a reduction of approximately 63%. Simulation and physical robot experiments further demonstrated that the method enabled closed-loop whole-body shape control and demonstrated robustness against visual noise, partial occlusion, and changes in mechanical properties, highlighting the advantages of deep learning for high-dimensional full-body shape modeling [79].

Overall, data-driven inverse modeling for soft robots has expanded beyond static input–output mappings to include dynamic relationships. These methods reduce reliance on explicit analytical inverse models by learning the relationships between desired outputs and actuation inputs. However, inverse models based primarily on static mappings provide limited information about the temporal evolution of system states. Forward dynamics modeling offers a complementary approach by learning the evolution of system dynamics to predict future states.

### 4.2. Forward Dynamics Modeling Based on Data-Driven Methods

Unlike the data-driven inverse models discussed above, forward dynamic models focus on predicting the future evolution of robot states from the current system state and actuation inputs [80]. An accurate forward model not only describes the dynamic evolution of a soft robot but also supports trajectory optimization and closed-loop control. However, high-fidelity continuum dynamic models are typically computationally expensive, limiting their practical use in dynamic prediction and control. Consequently, constructing efficient approximate dynamic models using data-driven methods has gradually become an important modeling approach.

Thuruthel et al. employed a NARX recurrent neural network to learn a forward dynamic model of a soft robot and combined it with single-shooting trajectory optimization to address the high computational complexity of analytical dynamic models, which limits their applicability to predictive control. The learned model required only 63 ms to perform a 2 s dynamic prediction, whereas the corresponding Piecewise Constant Strain (PCS) dynamic simulation required 523 s. The model achieved a mean reaching error of approximately 18 mm and was further extended to a four-segment soft robot, highlighting the potential of neural-network-based dynamic models to achieve efficient prediction while maintaining accuracy and scalability to more complex robotic structures [81]. Building on this work, Thuruthel et al. further employed a recurrent neural network to learn the forward dynamics, generated training data using trajectory optimization, and learned a closed-loop control policy through supervised learning to overcome the sensitivity of open-loop prediction to disturbances and model errors. When applied to a physical robot, the resulting closed-loop controller achieved a mean tracking error of 9 ± 8 mm and a tracking error of 22 ± 22 mm after an unmodeled 105 g payload was added to the end effector. Moreover, approximately 2 h of real-robot data were required to construct the complete closed-loop controller, indicating the potential of learned dynamic models to support robust closed-loop control with relatively limited physical training data [82].

Although recurrent neural networks can effectively approximate complex nonlinear dynamics, their internal representations remain largely black-box and lack an explicit dynamical structure. To improve the structural interpretability and representation of learned dynamics, subsequent studies explored Koopman-operator-based modeling. Bruder et al. employed Koopman operator theory for data-driven system identification of soft-robot dynamics. By lifting the nonlinear dynamics into a higher-dimensional space and identifying a linear representation from the lifted data, the method provides a more structured representation of the underlying nonlinear dynamics. Experiments showed that the Koopman model achieved an average normalized root mean square error (NRMSE) of 2.1%, substantially lower than the 4.5% obtained with a neural network, 4.8% with an NLARX model, and 4.5% with a nonlinear Hammerstein–Wiener model. In addition, the Koopman approach yields an explicit state space representation, demonstrating its advantages in both prediction accuracy and structured representations suitable for model-based control [83].

Overall, data-driven forward dynamics modeling for soft robots has expanded beyond prediction accuracy and computational efficiency to consider nonlinear dynamics representation, model interpretability, and applicability to control. Koopman-based methods [84] approximate nonlinear dynamics through linear representations in a lifted space, providing structured models for prediction and control. However, their performance depends strongly on the choice of lifting functions, highlighting the need for more interpretable and systematically constructed model representations rather than improvements in prediction accuracy alone.

### 4.3. Soft Robot Modeling Based on Physics and Data Fusion

The studies discussed above demonstrate that data-driven methods can substantially improve the prediction efficiency and computational efficiency of complex nonlinear dynamics in soft robots. However, as the scope of model applications continues to expand, focusing solely on predictive performance is no longer sufficient to meet more demanding modeling requirements. Neural networks generally lack explicit physical interpretability, whereas Koopman-based methods depend strongly on the design of lifting functions. Both limitations highlight the shortcomings of purely data-driven models in terms of physical consistency and theoretical guarantees. Accordingly, incorporating physical priors and structural constraints into data-driven models offers a promising route to improving physical consistency, interpretability, and generalizability, thereby moving soft-robot modeling beyond purely data-driven fitting toward closer integration of physics and data.

To reduce the dependence of Koopman-based methods on manually designed or empirically selected lifting functions [85], Shi and Karydis proposed ACD-EDMD, an Analytical Construction for Dictionaries of Lifting Functions within the Extended Dynamic Mode Decomposition framework. The method analytically constructs appropriate lifting functions by exploiting topological information from the robot configuration space or workspace and further provides provable completeness and convergence guarantees. In experiments on a soft pneumatic leg, ACD-EDMD achieved mean squared errors of 0.464 mm and 0.616 mm in the y and z directions, respectively, for predicting the complete motion trajectory. Although ACD-EDMD achieved prediction accuracy comparable to that of Sparse Identification of Nonlinear Dynamical Systems (SINDy), it substantially reduced the training time from 2.77 s to 0.08 s [86]. This study moves Koopman-based modeling beyond empirical dictionary selection toward structure-informed construction of lifting functions with theoretical guarantees, while improving computational efficiency without compromising prediction accuracy.

While some studies have focused on improving the model structure and theoretical foundations of data-driven approaches, another line of research retains physics-based models and leverages learning-based methods to enhance their computational efficiency and suitability for optimization. El Hussieny et al. used the Absolute Nodal Coordinate Formulation (ANCF) to establish inverse static relationships and subsequently employed a deep convolutional neural network to learn the resulting inverse quasi-static mapping, thereby addressing the computational complexity associated with solving inverse problems for high-fidelity large-deformation models [87]. Using data generated from the ANCF model, the network directly predicts the tip moments required to achieve a desired end-effector pose, enabling rapid approximate solutions while retaining the physical information embedded in the underlying model. This approach demonstrates the potential of combining physics-based modeling with data-driven surrogate models to improve computational efficiency without discarding physically grounded system representations. Going a step further, Hu et al. proposed ChainQueen, a fully differentiable physics-based simulator for soft robots based on the Moving Least Squares Material Point Method (MLS-MPM), in which model, control, and structural parameters can be directly optimized through gradient-based methods. Its GPU implementation achieved approximately four- to nine-fold speedups over the baseline methods considered in the study and supported control optimization problems involving nearly 3000 decision variables [88]. Rather than replacing the underlying physical dynamics with a black-box approximation, ChainQueen embeds the physical simulation directly within a differentiable computational framework, thereby providing a unified mechanism for gradient computation across system identification, control, and structural design.

Overall, data-driven dynamics modeling for soft robots has expanded beyond prediction accuracy and computational efficiency to address model structure, theoretical foundations, physical consistency, and applicability to control and optimization. The reviewed studies highlight the potential of integrating data, system structure, and physical knowledge to improve the representation of complex dynamics. These developments underscore the need for models that not only accurately predict system behavior but also support subsequent control and optimization.

Table 3 summarizes the main advances, performance metrics, and existing limitations of various AI-driven modeling paradigms for soft robots. In summary, different AI methods provide complementary capabilities for addressing distinct modeling challenges in soft robots. Feedforward neural networks and deep visual models exhibit strong nonlinear mapping capabilities, enabling them to bypass inverse kinematics and inverse statics problems that are difficult to solve explicitly, while further enabling high-dimensional full-body shape representations. Recurrent neural networks exploit past state information to capture temporal dependencies and are therefore better suited to describing hysteresis, material memory, and complex dynamic evolution. Online learning can continuously update the model, allowing it to adapt to parameter variations and unknown environmental conditions. Furthermore, Koopman-based methods obtain structured dynamic representations through lifting nonlinear dynamics into higher-dimensional spaces, while analytical dictionary construction, physics-based surrogate models, and differentiable simulation further introduce structural priors and physical knowledge into the modeling process. Taken together, the core value of AI methods does not lie in replacing conventional modeling with a single black-box model, but rather in providing targeted modeling tools for the distinct challenges of soft robots, including strong nonlinearities, complex dynamic behaviors, pronounced dependence on environmental conditions, and the high computational cost of physics-based models.

## 5. AI-Driven Methods for Addressing Control Challenges in Soft Robotic Systems

Accurate modeling provides the basis for describing and predicting soft robot behavior, whereas control involves converting system states, model information, and task objectives into effective control inputs. Previous studies have explored different control strategies to improve soft robot performance. Al-Ibadi et al. combined neural network and proportional control to enhance tracking accuracy and response speed [89], Andrikopoulos et al. developed nonlinear PID-based control to improve tracking and disturbance rejection [90], and Diteesawat et al. introduced an enhanced closed-loop PI strategy to improve dynamic control performance [91]. Owing to continuous deformation, complex environmental interactions, varying loads, and modeling uncertainties, soft robot control typically relies on closed-loop feedback to dynamically adjust control actions [92,93]. AI methods can further exploit closed-loop control, online adaptation, and task-oriented optimization by learning control mappings, using learned models for predictive optimization, or updating control policies from interaction data, thereby reducing reliance on accurate physical models [94,95]. Consequently, AI provides an important pathway toward improving adaptability and autonomous control performance under complex operating conditions.

### 5.1. Soft Robot Control Based on Inverse Mapping Learning

Early data-driven control studies primarily focused on learning inverse mappings between desired robot behaviors and actuation inputs, thereby avoiding the computational burden of solving analytical inverse models online. To address the nonlinearity, hysteresis, and feedback delays associated with pneumatic soft robots, Queißer et al. employed a Constrained Extreme Learning Machine (CELM) to approximate the inverse quasi-static equilibrium mapping between robot segment lengths and actuation pressures, integrating the learned model with a low-gain PID feedback controller. The CELM achieved prediction errors of 36.5, 61.7, and 54.6 mbar for the three robot segments, respectively, all lower than the corresponding errors obtained using the linear model (52.7, 83.0, and 78.4 mbar), while accelerating the convergence of the control response through feedforward compensation [96]. This study demonstrated that learning-based inverse mapping could improve the transient response without requiring higher feedback gains, providing an early example of combining data-driven feedforward compensation with conventional feedback control. Giorelli et al. subsequently investigated neural network-based inverse static mapping for soft manipulator control. A feedforward neural network (FNN) was trained to map desired tip positions directly to cable tensions, enabling rapid computation of actuation commands from task-space targets without the need to solve analytical inverse models online [43]. García-Samartín et al. further extended direct learning approaches to a pneumatic soft robot by employing feedforward neural networks to learn mappings from target positions to bladder inflation times and from sensor measurements to estimated actuator states, respectively, which were combined with proportional feedback to form a closed-loop controller [97]. After training on approximately 700 real-robot samples, the method achieved a position error of approximately 4.59 mm, demonstrating that millimeter-level position control can be realized without explicitly constructing a complex physical model. Overall, direct inverse mapping-based approaches reduce dependence on analytical models and enable computationally efficient implementation in online control. However, their performance depends heavily on the diversity and coverage of the training data, potentially limiting their generalization to substantially different operating conditions.

Collectively, these studies illustrate the use of learned inverse mappings as an alternative to analytical inverse models for soft robot control. CELMs and feedforward neural networks learn mappings from desired task-space outputs or system states to actuation inputs, reducing reliance on explicit analytical models and supporting efficient control-input estimation. Such methods are useful when analytical inverse models are difficult to derive, and the input–output relationships remain relatively stable. However, static or quasi-static mappings do not explicitly account for temporal dependencies and may therefore have limited capability to capture dynamic effects, including hysteresis and dynamic coupling, or to generalize across changing operating conditions. These limitations motivate the development of control-oriented dynamic models that capture the temporal evolution of soft robotic systems and can be integrated into predictive control frameworks.

### 5.2. Learning Control Models and Optimizing Control Algorithms

Compared with directly learning inverse mappings, another line of research leverages data to learn predictive forward models and subsequently integrates these models with optimization-based control frameworks. In this way, data-driven models can provide predictive capabilities while enabling efficient control optimization. To capture fabrication imperfections and other real-world effects that are difficult to represent using analytical models, Bern et al. employed a differentiable feedforward neural network to learn a forward mapping from control inputs to end-effector positions and utilized the gradients of the learned model to optimize control inputs [98]. The model trained on real-world data achieved mean tracking errors ranging from 0.14 to 0.22 cm across three trajectory-following tasks, substantially lower than the 0.62–0.92 cm obtained using the model trained on simulation data. These findings highlight the ability of experimentally trained forward models to account for physical effects not adequately represented in idealized simulations while facilitating gradient-based control optimization.

To construct dynamic models better suited for online control and optimization, Bruder et al. and Wang et al. further developed a relatively systematic line of research based on Koopman-MPC. Bruder et al. first employed the Koopman operator to lift the nonlinear dynamics of a soft robot into a higher-dimensional space and construct an approximately linear dynamical representation that could be directly incorporated into MPC. The mean trajectory-tracking error was reduced from 2.45 cm with a linear state-space MPC controller to 1.26 cm, demonstrating the feasibility of using data-driven linear representations for model predictive control of soft robots [99]. However, existing Koopman-based control methods for soft robots still exhibit limitations in high-speed dynamic motions and large-deformation regimes. Haggerty et al. further developed a Koopman operator-based dynamic control approach, which learns the robot dynamics, including inertial effects, to obtain a dynamic representation suitable for control optimization and integrates it with a closed-loop control strategy to achieve high-speed motion control of continuum soft robotic manipulators. Compared with conventional quasi-static control methods, this approach achieved approximately a 10-fold increase in motion speed and a 40-fold increase in acceleration, further extending the applicability of data-driven approaches to dynamic soft robot control [100]. Subsequently, LASSO regularization and projection were introduced to improve model sparsity and prediction stability, respectively. The resulting linear Koopman model achieved a mean prediction error of 1.92 cm, compared with 4.74 cm for the linear state-space model and 3.18 cm for the NARX model. Consequently, the mean tracking error of Koopman operator-based model predictive control (K-MPC) was further reduced to 0.46 cm, with an online computation time less than 0.083 s [101].

Subsequent studies extended Koopman-based approaches to accommodate varying operating conditions and improve the integration of model and controller design. To address dynamic variations caused by variations in payload, the load was further incorporated as an augmented state and estimated online using an observer, reducing the RMSE under variable loading conditions from 31.9 to 21.4 mm [102]. Building on this framework, Wang et al. further focused on the joint design of the Koopman model structure and controller. They constructed a KDTD model by extending the measurement coordinates with derivative and time-delay coordinates and employing SINDy for basis-function selection. An incremental model predictive control with dynamic constraints (IMPCDC) controller was then developed based on the Koopman model. This approach reduced the short-term prediction RMSE from 2.282° to 1.434° and, in the minimum-jerk trajectory-tracking task, reduced the control RMSE from 7.209 with conventional MPC to 1.700 [103]. Collectively, these studies illustrate the progression of Koopman-based MPC from initial demonstrations of feasibility toward improved prediction accuracy, computational efficiency, adaptability to varying operating conditions, and closer integration of model and controller design.

To enhance control robustness under complex dynamic conditions, researchers have investigated strategies that integrate physics-based priors with data-driven learning. To mitigate performance degradation arising from hysteresis, plastic deformation, nonlinear stiffness, and other nonlinear effects, Johnson et al. integrated a first-principles model with deep residual learning. A residual deep neural network (DNN) was employed to compensate for discrepancies between the physics-based model predictions and actual system dynamics, with the resulting hybrid model embedded in a nonlinear evolutionary model predictive control (NEMPC) framework [104]. With the learned error model incorporated, the mean tracking error was reduced from 0.378 to 0.182 rad. This approach exploits physics-based prior knowledge to preserve physically meaningful system dynamics and control constraints, while data-driven error compensation improves the controller’s adaptability to unmodeled dynamics, reflecting a broader transition in AI-enhanced control from purely data-driven approaches to hybrid control frameworks that integrate physical constraints with data-driven learning.

In addition to learning control-oriented models, AI-based methods can approximate computationally intensive control laws to reduce online computational burden. To alleviate the computational cost of solving the state-dependent Riccati equation (SDRE) online, Samadi Khoshkho et al. employed multilayer perceptron (MLP) neural networks to approximate the SDRE controller and subsequently applied knowledge distillation (KD) to compress the teacher network from 26,953 trainable parameters to a distilled network with 4130 trainable parameters [105]. In this way, the computationally expensive control law, which would otherwise require online SDRE computation, was approximated by a lightweight neural network, demonstrating that machine learning can serve not only as a tool for constructing control-oriented models but also as a computational accelerator for conventional optimal control algorithms.

Overall, learning-enhanced control for soft robots extends beyond replacing analytical models with data-driven alternatives and encompasses approaches addressing different control requirements. Differentiable neural models enable gradient-based control optimization, while Koopman representations approximate nonlinear dynamics using linear models in a lifted space to facilitate model-based optimization. Hybrid physics–data-driven models incorporate physical priors to support the control of nonlinear dynamics, whereas control-law learning can reduce reliance on repeated online optimization. Collectively, these approaches highlight the importance of considering not only prediction accuracy but also control performance, computational cost, adaptability, and optimization efficiency, contributing to the development of more effective control strategies for soft robots operating in complex dynamic environments.

### 5.3. Reinforcement Learning-Based Autonomous Control of Soft Robots

The studies discussed above mainly provide computationally efficient, control-oriented representations of soft robotic systems by learning kinematic mappings or constructing data-driven dynamic models. Beyond these approaches, researchers have also sought to reduce the reliance of control processes on explicit models. In this regard, reinforcement learning (RL) directly learns state-to-action policies through continuous interaction between the robot and its environment, providing an alternative control paradigm for complex soft robotic systems.

To address the high training burden of reinforcement learning on physical soft robots, Li et al. employed a low-fidelity simulator based on a PCC model to pre-train a Q-learning controller and subsequently fine-tuned it using real-world robot data. Despite the limited accuracy of the simulator, this approach reduced the required real-world data collection time by approximately 85% and substantially accelerated the convergence of the control policy. The results demonstrate that even a low-fidelity simulator can serve as an effective prior knowledge source for improving the sample efficiency of reinforcement learning on physical soft robots [106]. To further address multi-degree-of-freedom control and safe exploration, Ji et al. proposed a shielded multi-agent deep Q network (MADQN), in which different degrees of freedom were assigned to independent agents, while a shielding scheme dynamically constrained the feasible action space. Compared with a single-agent DQN, the proposed method reduced the training time from approximately 150 min to 50 min. With the shielding mechanism enabled, the root-mean-square (RMS) tracking errors for three different trajectories were reduced to approximately 0.24–0.31 mm, while high control accuracy was maintained under external loads and collision disturbances [107]. The method therefore simultaneously improves learning efficiency, exploration safety, and robustness to complex external disturbances.

As reinforcement learning has been increasingly applied to soft robot control, research has expanded from improving training efficiency and exploration safety toward coordinated control of high-degree-of-freedom systems and physical interaction tasks in complex environments. Ju et al. proposed an RL-based coordinated control framework, in which a Proximal Policy Optimization (PPO) policy trained in simulation and combined with proprioceptive feedback to achieve coordinated control of multiple degrees of freedom in multi-segment soft robots, thereby addressing the difficulty of manually designing control policies for high-dimensional soft robotic structures [108]. To address dynamic uncertainties and the challenges of force control arising from interactions between soft robots and their environments, Alessi et al. combined deep reinforcement learning with a dynamic Cosserat rod model and domain randomization to achieve sim-to-real transfer of closed-loop pose/force control. The approach was validated in dynamic pushing tasks, demonstrating the capability of reinforcement learning to handle complex physical interactions [109].

Overall, reinforcement learning (RL) for soft robot control has expanded to address sample efficiency, exploration safety, coordinated control of high-degree-of-freedom systems, and complex environmental interactions. Existing studies have shown that simulation-based pretraining can reduce the need for physical robot interactions, while multi-agent learning and safety-constrained approaches can support coordinated control and mitigate exploration risks, respectively. RL-based coordinated policies have also demonstrated their potential for controlling highly redundant soft robotic systems. The value of RL therefore extends beyond serving as an alternative to explicit model-based control, as it enables control policies to be learned through interaction, particularly for behaviors that are difficult to describe analytically. Nevertheless, achieving sample-efficient, safe, and stable control under limited real-world data and complex interaction conditions remains an important challenge.

As summarized in Table 4, diverse AI-based control paradigms possess distinct strengths, key performance metrics, and limitations for soft-robot control. In summary, the control challenges of soft robots arise primarily from the system uncertainties introduced by continuous deformation, strongly nonlinear material behaviors, and complex environmental interactions. These characteristics make it difficult for conventional model-driven approaches to simultaneously satisfy the requirements of accurate, efficient, and robust control. AI methods enhance the capability of control systems to represent and adapt to complex nonlinear dynamics through data-driven representations, dynamic prediction, and policy optimization. At the stage of control mapping learning, inverse mapping learning directly learns the relationship between target states and actuator inputs, thereby reducing the computational cost of solving inverse model problems and improving control response efficiency. Learning-enhanced model-based control further integrates AI into control-oriented models and optimization processes, improving control performance by constructing system representations that are better suited for prediction and optimization. Reinforcement learning, in turn, autonomously learns control policies from environmental feedback, enabling robots to adapt to unknown tasks and complex interaction environments. These approaches operate at different stages of the control pipeline, including control input generation, dynamic prediction, and policy decision-making, and collectively promote the development of intelligent control frameworks for soft robotic systems. Compared with conventional approaches, these methods offer key advantages in handling complex nonlinearities, reducing dependence on accurate models, improving computational efficiency, and enhancing adaptability and robustness under uncertainties and environmental changes. Nevertheless, current AI-driven control methods still face challenges related to data efficiency, generalization ability, physical consistency, and safe deployment in real-world environments. Future research should further integrate physical priors, online learning, and autonomous decision-making mechanisms, while promoting closer coordination among modeling, control, and planning, ultimately enabling more reliable and autonomous intelligent soft robotic systems.

## 6. AI-Driven Methods for Addressing Motion Planning Challenges in Soft Robots

Following advances in soft robot modeling and control, motion planning constitutes a critical step toward translating system-level research into practical applications by generating executable trajectories tailored to task objectives and environmental conditions. Compared with modeling and control, motion planning for soft robots is more closely associated with real-world tasks and must simultaneously address high kinematic redundancy, high-dimensional search spaces, environmental obstacles, and multiple constraints [110]. Through data-driven learning, autonomous decision-making, and global optimization, AI-based methods offer promising approaches to reducing reliance on accurate models and manually designed planning strategies while enhancing planning efficiency and adaptability to complex environments [111].

### 6.1. Learning from Demonstration-Based Motion Planning for Soft Robots

To address the challenges of motion planning for soft robots, AI methods provide promising solutions by learning motion patterns from demonstrations and data. Among these approaches, learning from demonstration (LFD) learns motion patterns from human demonstrations and enables robots to generate executable trajectories based on human experience, thereby reducing the difficulties caused by high redundancy, model uncertainty, and labor-intensive manual planning in soft robots. Wang et al. addressed the motion-planning challenges of multi-segment IPMC soft robots by employing Gaussian Mixture Models (GMMs) and Gaussian Mixture Regression (GMR) to learn from a small number of demonstrated trajectories, while a Jacobian-based inverse kinematic model was used to generate control commands [112]. Experiments demonstrated that smooth trajectories could be generated using only a limited number of demonstrations, enabling a six-segment flexible robot to successfully navigate through a narrow keyhole and reducing the dependence of the planning process on accurate models and computationally expensive search procedures. Building on this approach, Seleem et al. further addressed the limited generalization capability of learned trajectories under new goals and environmental changes by introducing Dynamic Movement Primitives (DMPs), together with path-integral optimization, black-box optimization, and an obstacle-coupling mechanism. The resulting framework enabled trajectory adaptation to new initial and goal states while avoiding static and moving obstacles [113]. These advances illustrate the progression of learning from demonstration beyond trajectory reproduction toward motion planning with improved goal generalization and environmental adaptability.

Seleem et al. subsequently conducted a systematic series of studies on the application of learning from demonstration to motion planning for complex continuum robots. First, they extended DMP-based learning from position trajectories to full pose planning by learning trajectories of the robot tip position and orientation and integrating them with the robot dynamic model and adaptive control. This enabled trajectory generalization to new initial and goal poses as well as stable tracking in obstacle-rich environments [114]. Subsequently, to address synchronization issues and orientation singularities arising from the separate representation of position and orientation, the authors introduced dual quaternions to provide a unified representation of rigid-body transformations and incorporated them into the DMP framework, thereby enhancing the consistency and robustness of full-pose motion generation [115]. Building on these developments, they further extended imitation-based planning beyond free-space motion toward tasks involving environmental interaction by integrating DMPs with dynamic impedance control. This enabled the robot to achieve compliant interaction while maintaining trajectory generalization capability and obstacle avoidance performance. Monte Carlo simulations were further conducted to assess the robustness of the overall system [116]. Collectively, this series of studies illustrates the evolution of learning from demonstration, progressing from position trajectory generation toward more sophisticated motion planning strategies that jointly consider full-pose motion, coordinated motion generation, and environmental interaction.

In summary, learning from demonstration (LfD) for soft robot motion planning has expanded from trajectory imitation to motion adaptation under varying task and environmental conditions. Demonstration-based approaches use demonstrations to learn motion patterns and guide trajectory generation in highly redundant systems. Subsequent approaches based on dynamic movement primitives (DMPs) supported goal adaptation and obstacle avoidance, while related developments explored full-pose motion generation and compliant interaction during environmental contact. These methods leverage demonstrated experience to provide motion priors for planning in high-dimensional configuration spaces. However, their performance remains dependent on the quality and diversity of demonstrations, limiting generalization beyond the demonstrated conditions. Future research could further combine LfD with reinforcement learning and other learning-based decision-making methods to improve planning adaptability and autonomy in unfamiliar environments.

### 6.2. Reinforcement Learning and Intelligent Optimization-Based Planning for Soft Robots

Unlike learning from demonstration (LfD), which relies on existing demonstration trajectories, reinforcement learning and intelligent optimization autonomously generate motion plans based on environmental feedback, task objectives, and constraints. Reinforcement learning learns decision policies through interaction, while sampling-based optimization and intelligent search generate feasible trajectories under multiple constraints, reducing model dependence and improving planning in high-dimensional spaces. Accordingly, soft robot motion planning is evolving from motion policy learning and constrained path optimization toward integrated planning-control and safe autonomous planning in dynamic environments.

For motion policy learning, Li et al. applied Soft Actor-Critic (SAC) to dielectric elastomer-actuated underwater soft robots, directly learning state-to-action mappings without accurate dynamic models. The physical robot achieved stable locomotion at approximately 6 mm/s with a randomness level of 0.08, demonstrating the shift from model-based motion generation to data-driven policy learning [117]. However, complex path planning and obstacle avoidance were not considered. El-Hussieny and Hameed employed a deep Q-network (DQN) to learn growth and steering policies for soft growing robots. Approximately 75% of random-goal trials achieved positive cumulative rewards, enabling autonomous navigation in obstacle-rich environments [118]. These studies extend reinforcement learning from goal-directed motion to environment-aware navigation.

Beyond policy learning, sampling-based search and intelligent optimization improve path feasibility and constraint handling. Khan et al. proposed the Weighted Jacobian Rapidly-exploring Random Tree (WJRRT), integrating RRT sampling, Jacobian control, and path weighting for Cartesian-space planning of soft manipulators [119]. The physical robot successfully tracked planned trajectories with a task time of approximately 9.43 s, although whole-body collision and dynamic environments were only partially addressed. Zhang et al. incorporated constrained sampling, artificial potential fields, and adaptive tissue parameters into RRT* for flexible needle insertion [120]. Compared with conventional RRT, the path length decreased from 333 to 291 mm and obstacle clearance increased from 353 to 411 mm, demonstrating improved path efficiency and safety, although replanning under dynamic tissue deformation remains limited.

For structural and multi-constraint planning, Xu et al. combined A* search, structure-aware collision detection, and PID-ILC control for 3D obstacle avoidance of a soft manipulator, achieving collision-free motion with trajectory errors below 5 mm [121]. However, adaptability to whole-body deformation and dynamic obstacles remained limited. Xiao et al. combined Jellyfish Search (JS) with quintic polynomial trajectories to jointly optimize camera orientation, obstacle avoidance, actuation, and motion time, demonstrating continuous trajectory planning under multiple constraints [122].

Research has further progressed toward integrated planning–control. Jitosho et al. developed a goal-conditioned reinforcement learning framework in which a single policy directly generates control actions from the robot state and target. Combined with sim-to-real transfer and domain randomization, the approach enabled agile goal-directed motions without online dynamic-model optimization, advancing soft robots from sequential planning and control toward unified motion generation. Dynamic whole-body safety, however, was not explicitly addressed [123].

For safe autonomous planning in dynamic environments, Luo et al. proposed Safety-Constrained RRT by integrating whole-body safety constraints, RRT optimization, and dynamic obstacle updates. The method reduced path length by approximately 17–25% over conventional RRT [124]. Physical experiments achieved an average planning time of 8.06 s with a 5.48 mm end-effector error, while dynamic-obstacle tests achieved 4.64 s and 0.47 mm, respectively. This work extends soft robot planning from static path generation to whole-body safe planning in dynamic environments, although planning efficiency and model dependence remain challenges.

Overall, AI methods are shifting soft robot motion planning from reliance on human demonstrations and experience toward autonomous decision-making and global optimization. Reinforcement learning learns motion policies through interaction with the environment, making it particularly suitable for sequential decision-making under model uncertainty and complex environmental conditions, whereas intelligent optimization handles multiple constraints, including attitude, obstacle avoidance, actuation, and motion time, through global search. These two classes of methods enhance the autonomy and optimization performance of motion planning, respectively, although their performance remains limited by sample efficiency, reward design, and computational costs associated with optimization. Future research could further integrate learning and optimization mechanisms to improve the planning efficiency, robustness, and generalization capability of motion planning in complex dynamic environments.

In summary, motion planning for soft robots requires the joint consideration of morphological deformation, task objectives, and environmental constraints under continuous deformation, high-dimensional configuration spaces, and complex environmental interactions. AI-based methods provide alternative strategies for addressing these challenges by learning motion representations, optimizing planning policies, and reducing reliance on explicit analytical models or manually designed planning rules. Among these methods, learning from demonstration (LfD) extracts motion priors from expert demonstrations and supports trajectory reproduction and generalization under related task conditions. Reinforcement learning (RL), in contrast, learns state–action policies through interaction with the environment, allowing motion policies to be progressively refined through feedback. Thus, LfD and RL provide complementary paradigms that emphasize the reuse of demonstrated experience and interaction-based policy learning, respectively. Demonstration-derived policies or motion priors could further provide useful initialization for RL and reduce unnecessary exploration in high-dimensional spaces, while RL could potentially refine demonstrated behaviors through environmental feedback. In addition, intelligent optimization methods can incorporate multiple planning constraints and search for high-quality trajectories within complex solution spaces. Nevertheless, LfD remains dependent on the diversity and coverage of demonstrations and may generalize poorly to conditions beyond the demonstrated distribution, whereas RL continues to face challenges in reward design and the safety of exploration and deployment. A detailed comparison is provided in Table 5. Future research could further integrate demonstration priors, adaptive policy refinement, and physics-informed constraints to improve transferability, safety, and adaptability in soft-robot motion planning.

## 7. Conclusions

### 7.1. Current Advances and Remaining Challenges

Artificial intelligence offers promising approaches to addressing the challenges arising from continuous deformation, strongly nonlinear behavior, model uncertainty, and environmental coupling in soft robots. In modeling, neural networks and related methods can reduce reliance on complex analytical models while enhancing system representation and dynamic prediction. In control, data-driven methods and reinforcement learning can enhance control robustness and adaptability under model uncertainty and complex disturbances. In motion planning, learning from demonstration and intelligent optimization facilitate autonomous decision-making and computationally efficient planning. Collectively, these advances support the integration of modeling, control, and motion planning, laying the groundwork for autonomous operation of soft robots in complex environments.

Despite these advances, AI-driven soft robotic systems still face several key challenges. First, soft robots are affected by material variability, hysteresis, parameter drift, and environmental changes, while learning-based models typically rely on large amounts of high-quality data. Consequently, models and policies trained under limited operating conditions may generalize poorly to new payloads, environments, and robotic platforms. Second, although purely data-driven methods provide powerful nonlinear approximation capabilities, they often lack explicit physical constraints, whereas physics-based models generally incur high computational costs. Balancing prediction accuracy, physical consistency, and computational efficiency therefore remains a key challenge. Finally, reinforcement learning and autonomous planning methods face challenges associated with costly real-robot training, safe exploration, and computationally efficient decision-making. Moreover, modeling, control, and motion planning are still largely studied separately, and their integration into a coordinated system-level framework remains an open challenge.

### 7.2. Future Prospects

In view of the above limitations, future research on AI-driven soft robotics should further improve the generalization, physical consistency, safety, and cross-module coordination of learning-based methods, promoting a transition from task-specific optimization toward system-level autonomy in complex environments.

First, to improve the generalization of models and control policies across different materials, payloads, robotic platforms, and environmental conditions, future studies could explore data augmentation, transfer learning, domain adaptation, and online learning. Dynamic spatiotemporal graph generative adversarial networks (DSTG-GANs) can learn complex dynamic nonlinear spatiotemporal dependencies and generate corresponding scenarios, providing a new approach to representing the spatiotemporal coupling of distributed deformation, actuation, and sensing states in soft robots [125]. Investigating their application for data augmentation could enable the generation of representative scenarios covering diverse payloads, actuation conditions, and environmental interactions, thereby expanding operating-condition coverage and potentially improving model generalization. Such strategies could improve adaptability to different robot configurations while reducing the need for extensive data collection and repeated retraining.

Second, greater integration of physical priors and data-driven learning is needed to balance physical consistency and computational efficiency. Koopman-based methods can provide structured representations of nonlinear soft-robot dynamics in higher-dimensional approximately linear spaces, supporting efficient prediction, system identification, and model-based control. Meanwhile, incorporating physical priors such as material properties, kinematic constraints, constitutive relations, and simplified analytical models could improve physical consistency and data efficiency. Further integration of Koopman representations with physics-informed learning may therefore offer a promising direction for efficient and physically consistent soft-robot modeling.

Third, to overcome the high training cost, unsafe exploration, and decision-making efficiency limitations of reinforcement learning and autonomous planning, more sample-efficient and safety-aware learning methods should be developed. Model-based reinforcement learning, offline learning, sim-to-real transfer, and safety-constrained learning could reduce the dependence on real-robot data and mitigate training risks, thereby improving autonomous decision-making and safe operation in complex dynamic environments.

Finally, as modeling, control, and motion planning are often investigated separately, future research should emphasize their system-level coordination. Learning-based models could provide online state estimation and dynamic predictions to support control and planning, while feedback could be used to update models and policies. Adaptive dynamic scenario clustering algorithms (ADSCA) can identify representative scenarios while preserving key spatiotemporal correlations, offering a potential approach to scenario selection under complex operating conditions [126]. Their application to scenario selection for model prediction, motion planning, and control optimization could be explored to reduce scenario redundancy and potentially alleviate computational demands. Reinforcement learning and intelligent optimization could support the coordination of high-level planning and low-level control. Together, these approaches could facilitate a closed-loop process of prediction, decision-making, control, and feedback updates, supporting the transition from stage-specific intelligence toward system-level autonomy in soft robots.

## Figures and Tables

**Table 1:** Inclusion and exclusion criteria used for literature selection.

Criterion	Inclusion Criteria	Exclusion Criteria
Publication year	Publications between 2015 and 2026	Publications outside this period
Research subject	Soft robots, continuum robots, and compliant robotic systems falling within the defined scope	Rigid robots and other robotic systems outside the defined scope
Research domain	Modeling, control, or motion planning of soft robotic systems	Studies outside these three research domains
Research methodology	AI, ML, DL, RL, neural networks, or related data-driven approaches	Studies without an AI/data-driven component

**Table 2:** Key Challenges in Modeling, Control, and Motion Planning of Soft Robots.

Challenge Source	Challenges of Modeling	Challenges of Controlling	Challenges of Planning
Continuous deformation and infinite degrees of freedom	Developing compact and accurate representations to describe high-dimensional continuous deformations while reducing model complexity	Difficulty in establishing accurate mappings from desired motions to actuation inputs	Difficulty in planning motions considering whole-body shape variations
Nonlinear and uncertain material behaviors	Accurately modeling coupled material and geometric nonlinearities under complex deformation conditions	Nonlinear and hysteretic input–response relationships reduce control accuracy	Deformation-induced geometric changes increase the difficulty of collision detection and trajectory generation
Time-dependent material properties and parameter uncertainties	Capturing hysteresis, creep, stress relaxation, and material variations for reliable long-term prediction	Control parameters may lose effectiveness, resulting in unstable responses and accumulated control errors	Model prediction uncertainties reduce trajectory execution reliability
Actuation–structure–environment coupling	Establishing integrated models that describe interactions among actuation, deformation, and environmental constraints	Difficulty in accurate online state estimation and maintaining closed-loop control stability	Environmental interactions modify robot configurations and constraints, making feasible path searching more challenging

**Table 3:** Advances of AI-based modeling methods for soft robots.

AI-Based Modeling Paradigm and References		Representative Methods and Studies	Key Metric and Result (Note: “—” Indicates That the Data Were Not Reported.)	Main Advances	Current Limitations
Learning-based inverse modeling	[43]	FNN	positioning error: 7.35 mm inference time: 0.114 ms	Learning complex actuator–configuration mappings to simplify inverse modeling and improve prediction efficiency	Strong data dependency and limited physical interpretability
	[77]	NN modified Elman NN	—		
	[78]	LWPR	—		
High-dimensional state representation learning	[79]	CNN	image-mask error: 0.0133 reduction: ~63%	Whole-body morphological learning for improved deformation modeling beyond low-dimensional representations	Sensitive to sensing quality and data diversity
Data-driven forward dynamics modeling	[81]	NARX-RNN	prediction time: 63 ms reaching error: ~18 mm	Nonlinear dynamics learning for efficient prediction and reduced computational cost	Black-box characteristics and limited generalization
	[82]	RNN	tracking error: 9 ± 8 mm 105 g payload: 22 ± 22 mm		
Structured nonlinear dynamics modeling	[83]	Koopman	NRMSE: 2.1%	Structured dynamics representations for accurate, interpretable, and transparent nonlinear modeling	Dependence on suitable lifting functions and model construction
	[84]	Koopman-based method	—		
	[86]	ACD-EDMD	MSE (y/z): 0.464/0.616 mm training time: 0.08 s		
Physics- informed modeling	[87]	ANCF-CNN	—	Physics–data integration for efficient, physically consistent, and theoretically reliable modeling	Requires physical priors and may involve higher modeling complexity
	[88]	ChainQueen (MLS-MPM)	computation speed: 4–9× faster than baselines decision variables: ~3000		

**Table 4:** Advances of AI-based controlling methods for soft robots.

AI-Based Modeling Paradigm and References		Representative Methods and Studies	Key Metric and Result (Note: “—” Indicates That the Data Were Not Reported.)	Main Advances	Current Limitations
Data-driven inverse mapping	[96]	CELM	prediction error: 36.5/61.7/54.6 mbar	Learning complex state–actuation mappings to bypass difficult analytical inverse solutions and improve control efficiency	Data dependence and limited generalization; mainly suited to static or quasi-static mappings
	[43]	FNN	—		
	[97]	FNN-based inverse mapping	position error: ~4.59 mm		
Learning-based forward modeling and predictive control	[98]	Differentiable FNN	tracking error: 0.14–0.22 cm	Learning predictive system dynamics to improve trajectory tracking, computational efficiency optimization, and adaptability to changing operating conditions	Performance depends on training-data coverage and may degrade under complex dynamics
	[99]	Koopman-MPC	tracking error: 2.45 → 1.26 cm		
	[100]	Koopman-based dynamic control	speed: ~10× acceleration: ~40×		
	[101]	Koopman K-MPC	prediction error: 1.92 cm tracking error: 0.46 cm computation: <0.083 s		
	[102]	Load-augmented Koopman model	RMSE: 31.9 → 21.4 mm		
	[103]	KDTD + SINDy + IMPCDC	prediction RMSE: 2.282° → 1.434° control RMSE: 7.209 → 1.700		
Physics- informed hybrid control	[104]	Residual DNN + physics-based model + NEMPC	tracking error: 0.378 → 0.182 rad	Learning nonlinear state evolution to enable efficient dynamic prediction and reduce the computational cost of high-fidelity models	Requires physical priors and increases modeling complexity
Learning-based control-law approximation Learning-based control-law approximation	[105]	MLP + Knowledge Distillation	trainable parameters: 26,953 → 4130	Constructing structured dynamic representations to improve nonlinear modeling accuracy, interpretability, and model transparency	Learned approximation may provide limited adaptability outside the training domain
Reinforcement learning-based autonomous control	[106]	Q-learning	real-world data collection: ↓ ~85%	Integrating physical knowledge with data-driven learning to improve modeling efficiency, physical consistency, and theoretical reliability	High training cost, safety concerns, and sim-to-real gap
	[107]	Shielded MADQN	training time: ~150 → 50 min RMS error: 0.24–0.31 mm		
	[108]	PPO	—		
	[109]	DRL + Cosserat rod model + domain randomization	—		

Footnotes: Left-right arrows show value transitions from left to right; Downward arrows indicate the magnitude of reduction.

**Table 5:** Advances of AI-based planning methods for soft robots.

AI-Based Modeling Paradigm and References		Representative Methods and Studies	Key Metric and Result (Note: “—” Indicates That the Data Were Not Reported.)	Main Advances	Current Limitations
Learning from demonstration-based planning	[112]	GMM + GMR	—	Learning motion patterns from demonstrations to reduce high-dimensional search and improve trajectory generalization and environmental adaptability	Dependence on demonstration quality and diversity; limited adaptation to unseen tasks
	[113]	DMP + optimization	—		
	[114]	Full-pose DMP	—		
	[115]	Dual-quaternion DMP	—		
	[116]	DMP + impedance control	—		
Reinforcement learning-based motion policy learning	[117]	SAC	speed: ≈6 mm/s randomness level: 0.08	Learning state–action policies to reduce model dependence and enable autonomous motion and navigation	High training cost; limited generalization and path-planning capability
	[118]	DQN	positive-reward trials: 75%		
Sampling-based and intelligent optimization planning	[119]	WJRRT	task time: 9.43 s	Improving path feasibility, safety, obstacle avoidance, and multi-constraint optimization	Model dependence; limited whole-body and adaptive planning capability planning
	[120]	Improved RRT*	path length: 333 → 291 mm clearance: 353 → 411 mm		
	[121]	A* + PID-ILC	trajectory error: <5 mm		
	[122]	JS	—		
Integrated planning and control	[123]	Goal-conditioned RL	—	Unifying planning and control within a single policy to enable autonomous motion generation	High training cost; limited interpretability and complex-environment validation
Safe autonomous planning in dynamic environments	[124]	Safety-Constrained RRT	path length: ↓17–25% planning time: 8.06 s error: 5.48 mm dynamic: 4.64 s/0.47 mm	Enabling whole-body safety planning and online path updating under dynamic obstacles	Limited planning efficiency and unknown-environment adaptation; model dependence

Footnotes: Left-right arrows show value transitions from left to right; Downward arrows indicate the magnitude of reduction.

## Data Availability

No new data were created or analyzed in this study. Data sharing is not applicable to this article.

## Abbreviations

The following abbreviations are used in this manuscript:

AI	Artificial Intelligence
ANCF	Absolute Nodal Coordinate Formulation
CELM	Constrained Extreme Learning Machine
DMPs	Dynamic Movement Primitives
DQN	Deep Q-Network
DNN	Deep Neural Network
FNN	Feedforward Neural Network
GMMs	Gaussian Mixture Models
GMR	Gaussian Mixture Regression
IMPCDC	Incremental Model Predictive Control with Dynamic Constraints
JS	Jellyfish Search Algorithm
KD	Knowledge Distillation
K-MPC	Koopman Operator-Based Model Predictive Control
LFD	Learning from Demonstration
MADQN	Multi-Agent Deep Q Network
MLP	Multilayer Perceptron
MLS-MPM	Moving Least Squares Material Point Method
MPC	Model Predictive Control
NEMPC	Nonlinear Evolutionary Model Predictive Control
NRMSE	Normalized Root Mean Square Error
PAMs	Pneumatic Artificial Muscles
PCC	Piecewise Constant Curvature
PCS	Piecewise Constant Strain
PPO	Proximal Policy Optimization
RL	Reinforcement Learning
RMS	Root-Mean-Square
SDRE	State-Dependent Riccati Equation
SINDy	Sparse Identification of Nonlinear Dynamical Systems
